# A188 NATURAL HISTORY OF PERIANAL FISTULAS IN PATIENTS WITH ILEAL POUCH ANAL ANASTOMOSIS

**DOI:** 10.1093/jcag/gwae059.188

**Published:** 2025-02-10

**Authors:** M Fujiyoshi, J McCurdy

**Affiliations:** University of Ottawa, Ottawa, ON, Canada; University of Ottawa, Ottawa, ON, Canada

## Abstract

**Background:**

The natural history of perianal fistulas (PAF) in patients with ileal pouch-anal anastomosis (IPAA) remains poorly understood. Perianal fistulas can significantly impact the quality of life and clinical outcomes in patients who have undergone IPAA. To date, there is limited data available on the long-term progression, treatment responses, and overall outcomes of PAF in this unique patient population.

**Aims:**

The primary aim of this study is to report the clinical outcomes of PAF in patients with IPAA.

**Methods:**

We performed a retrospective, observation cohort study between 2000 and 2024. We included adults (>17 years) with an IPAA who developed a perianal fistula and a minimum of 1 year follow-up after PAF diagnosis. PAF disease activity was categorized on an annual basis according to the following disease activity patterns: remission, minimally active, relapsing-remitting, and chronic persistent. We also determined medication utilization, medication effectiveness and major adverse fistula outcomes (MAFO): a composite of hospitalization, surgical drainage of PAF and fecal diversion.

**Results:**

A total of 29 patients with an IPAA and PAF were identified: mean (SD) age 49.3 (13.2); 19 (65.5%) female, and 17 (63.0%) with complex fistulas radiologically at the time of diagnosis. After a mean (SD) follow-up of 9.7 years (7.4), 7 (25.0%) patients required fecal diversion, and 2 (7.1%) patients underwent pouch excision. Annual PAF disease patterns are reported in Figure 1. At the last date of follow-up, 26 (92.9%) patients achieved fistula remission. A total of 16 (55.2%) patients underwent treatment with an advanced therapy: 14 (87.5%) anti-TNF and 2 (12.5%) Risankizumab. After initiating a first advanced therapy, 10 (62.5%) patients achieved fistula remission at 12 months. Furthermore, 3 (18.8%) patients developed a MAFO after initiating their first advanced therapy: 3 (18.8%) underwent surgical drainage, 1 (6.3%) was hospitalized, and 1 (6.3%) underwent fecal diversion for PAF.

**Conclusions:**

This study demonstrated that the majority of patients with PAF in the setting of IPAA achieved fistula remission clinically, particularly after treatment with advanced therapies. Despite the high remission rates, a substantial subset of patients still required fecal diversion and pouch excision.

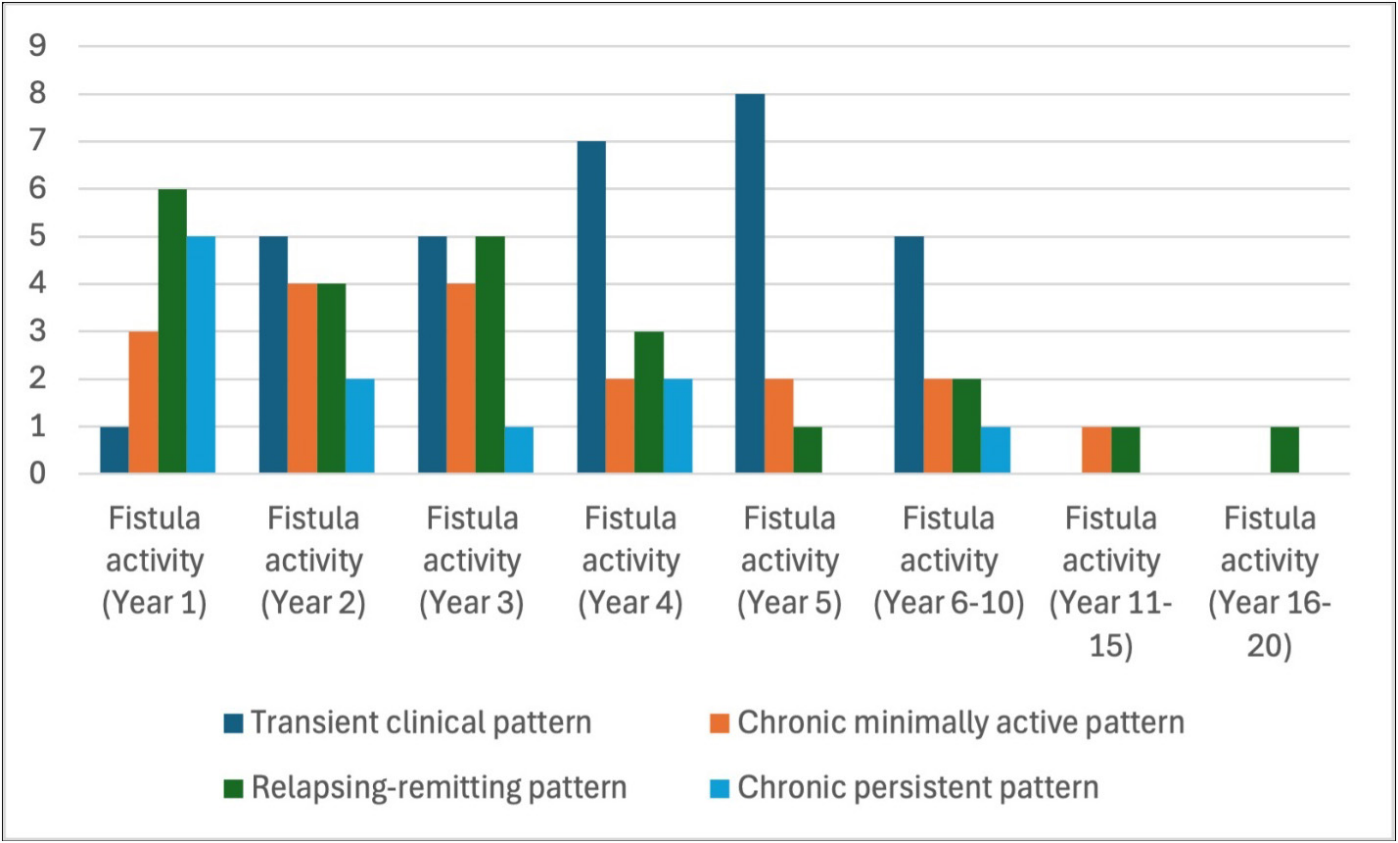

Annual perianal fistula disease patterns

**Funding Agencies:**

None

